# Molecular therapy for papillary craniopharyngioma: a multi-institutional analysis of practice patterns across the RAPID Consortium

**DOI:** 10.1007/s11060-026-05739-5

**Published:** 2026-08-14

**Authors:** Mark A. Damante, Ryan B. Juncker, Andrew S. Little, Jamie J. Van Gompel, Sandhya Palit, Donato R. Pacione, Garni Barkhoudarian, Walavan Sivakumar, Spiros Blackburn, Michelle Magaña Mendoza, James J. Evans, Carolina Benjamin, Joshua D. Palmer, Pierre Giglio, Daniel M. Prevedello, Kyle C. Wu, RAPID Consortium

**Affiliations:** 1 Department of Neurological Surgery, The Ohio State University Wexner Medical Center, Columbus, OH USA; 2 College of Medicine, The Ohio State University, Columbus, OH USA; 3 Department of Neurological Surgery, Barrow Neurological Institute, St. Joseph’s Hospital and Medical Center, Phoenix, AZ USA; 4 Department of Neurosurgery, Mayo Clinic, Rochester, MN USA; 5 Department of Neurosurgery, New York University, New York, NY USA; 6 Department of Neurosurgery, Pacific Neuroscience Institute, Providence St. Johns Medical Center, Santa Monica, CA USA; 7 Department of Neurosurgery, UT Health Houston, Houston, TX USA; 8 Neuroanalytics Center, Barrow Neurological Institute, St. Joseph’s Hospital and Medical Center, Phoenix, AZ USA; 9 Department of Neurosurgery, Jefferson University, Philadelphia, PA USA; 10 Department of Neurological Surgery, Miller School of Medicine, University of Miami, Miami, FL USA; 11 Department of Radiation Oncology, The Ohio State University Wexner Medical Center, Columbus, OH USA; 12 Division of Neuro-Oncology, Department of Neurology, The Ohio State University Wexner Medical Center, Columbus, OH USA

**Keywords:** Craniopharyngioma, Papillary craniopharyngioma, BRAF V600E, Targeted therapy, Radiation

## Abstract

**Purpose:**

Targeted therapy for BRAF V600E mutant papillary craniopharyngioma (PCP) has been rapidly accepted, though given the rarity of the tumor, there is limited guidance for appropriate use. Here, we retrospectively evaluated current practice patterns for the implementation of targeted therapeutics and their outcomes in the treatment of papillary craniopharyngioma (PCP).

**Methods:**

Practice patterns at the institutions in the Registry for Adenomas of the Pituitary and Related Disorders (RAPID) for treatment of BRAF V600E mutated PCPs using targeted therapy were evaluated. Baseline clinical and demographic variables were retrospectively recorded. Imaging response was evaluated. Dosing, treatment course, adverse events (AEs) and other therapeutic strategies employing BRAF/MEK inhibitors were studied. Adjuvant and/or salvage therapies, including radiation and surgery were recorded.

**Results:**

Most patients demonstrated at least partial response to BRAF and/or BRAF/MEK inhibition (13/19, 72.2%). No patients progressed through therapy. Grade 0–2 AEs occurred in 79% of cases, and nine (47.4%) patients eventually discontinued therapy. Radiation-sparing regimens demonstrated similar radiographic response rates and had smaller residual tumor volume after treatment completion (23.7 vs. 612.4 mm^3^, *p* = 0.040) compared to those receiving radiation. BRAF monotherapy demonstrated similar rates of response to therapy, but fewer Grade 3–4 AEs (0% vs. 28.5%) than dual therapy. Long-course therapy (> 4 cycles) resulted in radiographic response (*p* = 0.024) more often than a short-course (≤ 4 cycles), with similar AEs. Median follow-up duration was 19 months from surgery (range: 8–63).

**Conclusion:**

Critical insights into molecular therapeutic practice patterns for treatment of PCP were identified. Radiation-sparing, mono-therapeutic, and prolonged treatment duration are feasible options and warrant further study.

**Supplementary Information:**

The online version contains supplementary material available at https://doi.org/10.1007/s11060-026-05739-5.

## Introduction

Craniopharyngioma (CP) is a complex intracranial tumor thought to arise from remnants of the Rathke’s pouch [1, 2]. Though considered to be of low histological malignancy (World Health Organization [WHO] Grade 1), the lesion carries significant potential neuro-endocrinologic morbidity, with particular risk for progressive visual impairment, and may become recalcitrant to standard of care treatments [1, 2]. Whole exome analysis initially defined the molecular distinction between papillary (PCP) and adamantinomatous (ACP) types of CP [3]. The highly conserved BRAF V600E mutation defines PCP and represents a strategy for small molecule inhibitors, like other BRAF V600E mutation-harboring tumors [4–9]. 

The early success of BRAF and MEK inhibitors for PCP in clinical trials and institutional series demonstrates potential to substantially improve neuro-endocrinologic and oncologic outcomes [4, 8, 9]. The observed rapid and durable response to therapy has led to the exploration of different treatment strategies [4, 8, 10–12]. However, given the rarity of these tumors, it is difficult to conduct large, randomized clinical trials to establish the optimal treatment paradigm [13]. Here, we leverage the multi-institutional Registry for Adenomas of the Pituitary and Related Disorders (RAPID) Consortium with the primary aim to explore the current practice patterns for implementing BRAF and MEK inhibition for PCP and identify important considerations for future study.

## Methods

### Study design

The presented study is a multi-institutional retrospective cohort analysis of patients with surgically treated PCP receiving adjuvant BRAF and/or MEK inhibitors between January 1, 2017, and December 31, 2024. Collection of retrospective data was requested from the RAPID consortium following steering committee discussion regarding variables of interest, as previously described [14]. This study was approved by the local ethics committee and IRB at each participating institution with the overarching multi-institutional IRB governed by the IRB at Barrow Neurological Institute. The need for informed consent was waived by the multi-institutional IRB due to the entirely retrospective and minimal risk nature of this study. Clinical trial number: not applicable (retrospective study).

### Cohort

Adult patients with histopathologic diagnosis of PCP were included, including patients with BRAF V600E alterations identified via immunohistochemistry. All patients underwent upfront surgery at a participating RAPID center and received BRAF and/or MEK inhibitor targeted medical therapy during their treatment course. Patients with ACP and those not receiving BRAF and/or MEK inhibitors were excluded. Pediatric cases (age less than 18 years) were originally in the exclusion criteria, but no pediatric PCP cases treated with BRAF/MEK inhibitor therapy were identified. The total sample size per analysis is listed throughout.

### Variables

Demographic, medical history, treatment-related, and clinical course variables were determined by the RAPID steering committee prior to collection. Tumor size was recorded preoperatively, postoperatively prior to the initiation of medical therapy, and after the completion of medical therapy, and presented as volume (calculated using the maximum tumor diameters in the coronal, axial, and sagittal directions with the formula: [Volume = abc/2]; mm^3^). Images were evaluated locally at each participating institution with dimensions uploaded to the RAPID registry. Change in maximum volume from pre- to post-targeted therapy treatment was determined.

Therapies including repeat surgery and adjuvant or salvage radiation were recorded along with their temporal relation to targeted therapy treatment. BRAF and/or MEK inhibitor class and dose, treatment cycles, and time from surgery to medical therapy were collected. Short course therapy was considered four or fewer cycles. This cut-off was determined based on the presented cohort median (four cycles) and the recent phase I/II Alliance trial (NCT03224767; registered: 1/5/2018; completed: 9/22/2023), which employed four cycles of targeted therapy for all participants [8]. Cycles consisted of 3–4 week courses of targeted therapy (median 28 days), determined at the discretion of the treating institution.

The Response Assessment in Neuro-Oncology (RANO) 2.0 criteria determined response to therapy [15, 16]. Other outcomes collected included reason for therapy discontinuation (due to adverse events, due to progression through treatment, or other). Adverse events (AE) were graded using the Common Terminology Criteria for AEs (CTCAE) scale (1–5) [17]. 

### Progression free survival

Progression free survival (PFS) was defined as time of surgery to radiographic progression following completion of at least 1 cycle of targeted therapy. We compared the patients in this cohort receiving targeted therapy to patients in our recently published cohort of adult PCP patients managed surgically over the same study period who did not receive targeted therapy [18]. 14% of patients in this group received adjuvant or salvage radiation therapy in addition to surgical resection. Patients were excluded from the targeted therapy group if they progressed prior to therapy initiation. Patients were censored by the Kaplan-Meier methodology and compared by Log-rank test.

### Statistical analysis

Categorical variables were compared by chi-square test. The normality of distribution of continuous variables was determined by a Kolmogorov-Smirnov test, and compared by appropriate statistical tests, as described throughout the text. All statistical analyses were performed using the Statistical Package for Social Sciences (IBM SPSS Statistics, Version 22.0, Armonk, NY, IBM Corp). A two-tailed p-value of less than 0.05 was considered significant for all tests.

## Results

### Cohort

Of the 124 PCP patients cared for by RAPID Consortium institutions, we identified 19 who received targeted therapy during the study period. Median duration from surgery to last follow-up and initiation of medical therapy to last follow-up were 19 months (range, 8–63) and 12 months (range, 3–54), respectively.

Table 1 details the demographics and clinical course of the included cohort. Patients were a median of 52.5 (IQR: 39.0–61.0) years-of-age at surgery and 36.8% were male. The index treatment was considered the first surgery performed at the RAPID center at which the patient underwent medical therapy treatment. Six patients (31.6%) had undergone a previous surgery and/or radiation treatment in the remote past at an outside hospital (non-RAPID center) prior to the index treatment at the respective RAPID center. Preoperative median tumor volume was 5737.2 mm^3^ (2374.3–7961.2 mm^3^). No patients underwent gross total resection (GTR). Eighteen patients (94.7%) underwent subtotal or partial resection, and one patient received biopsy only.

**Fig. 1:**
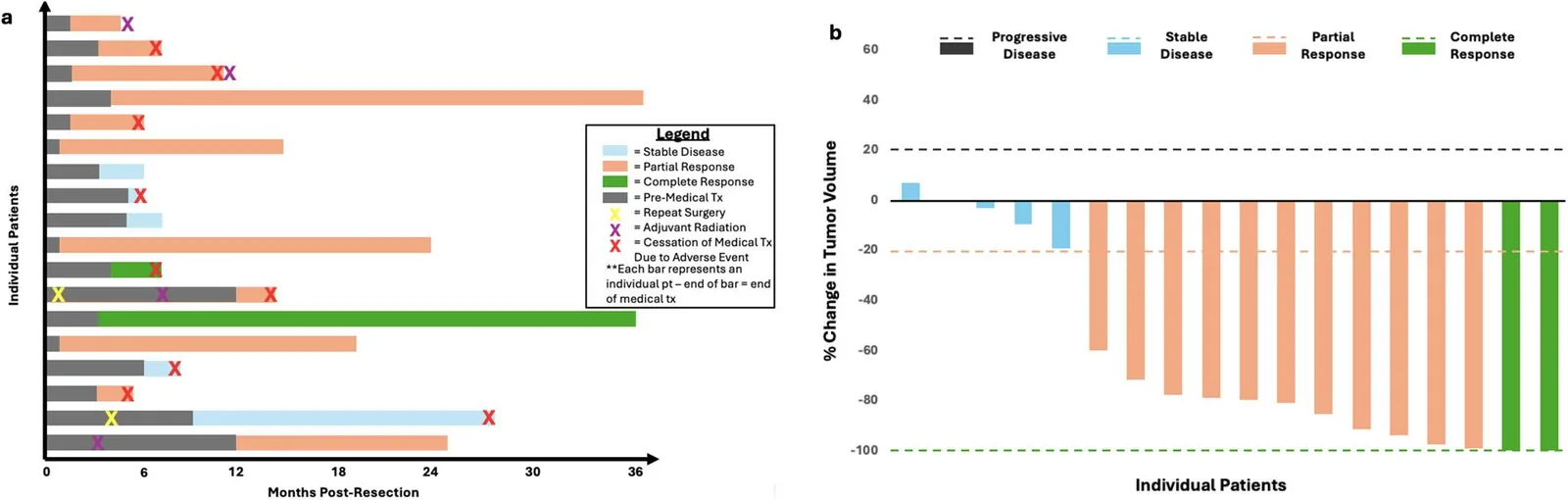
Panel **A** represents a depiction of the treatment course and response to therapy for each patient. Each bar represents an individual patient, with salvage therapies, timepoints, and response represented as shown in the legend. Time 0 represents the index surgery performed at the treating RAPID center. Panel **B** illustrates the pre- to post-medical therapy percent change in tumor volume and RANO 2.0 response to therapy class of each patient

Postoperatively (relative to the index RAPID center treatment), two patients (10.5%) progressed and underwent repeat resection prior to the initiation of targeted therapy. Four patients (21.1%) received adjuvant postoperative radiation, with two treated prior to targeted therapy initiation and two after medical therapy cessation despite no progression during therapy. Figure 1A details the treatment course and eventual response to therapy for each patient.

**Table 1:** Full cohort demographics, clinical course, medical therapy details, and outcomes

	PCP Med Tx Patients (*n* = 19)
Demographics and Prior Treatment	Absolute # (%) or Median (IQR)
Age at Surgery (years)	52.5 (39.0–61.0)
Male Gender	7 (36.8)
Remote hx of Previous Surgery at Outside Hospital	3 (15.8)
Remote hx of Previous Radiation Therapy at Outside Hospital	3 (15.8)
**Postoperative/Salvage Therapies**	
Repeat Surgery Prior to Medical Therapy	2 (10.5)
Adjuvant/Salvage Radiation Before the Initiation of Med Tx	2 (10.5)
Adjuvant/Salvage Radiation After the Initiation of Med Tx	2 (10.5)
**Medical Therapy Type and Dose**	
BRAF Inhibitor Tx Only	4 (21.1)
MEK Inhibitor Tx Only	1 (5.3)
Dual BRAF/MEK Inhibitor Tx	14 (73.7)
Number of Cycles of Therapy	4.0 (3.0–14.0)
Total Duration of Therapy (mos)	4.0 (2.0–17.0)
Time from Surgery to Initiation of Med Tx (mos)	3.0 (0.0–5.5)
**BRAF Inhibitor Dose (mg)**	
Vemurafenib*	960 (960–960)
Dabrafenib*	150 (50–300)
Encorafenib*	450 (150–450)
**MEK Inhibitor Dose (mg)**	
Trametinib*	2 (1–2)
Cobimetinib*	60 (60–60)
Binimetinib*	45 (30–45)
**RANO Response to Therapy**	*N* = 18
Complete Response to Med Tx	2 (11.1)
Partial Response to Med Tx	11 (61.1)
Stable Disease Through Med Tx	5 (26.3)
**Cessation of Therapy and Adverse Events**	
Stop Medical Tx due to Adverse Event	9 (47.4)
Stop Medical Tx due to Progression	0 (0.0)
Stop Medical Tx for Other Reasons	3 (15.8)
Grade 1 Adverse Event	5 (26.3)
Grade 2 Adverse Event	6 (31.6)
Grade 3 Adverse Event	3 (15.8)
Grade 4 Adverse Event	1 (5.3)
Grade 5 Adverse Event	0 (0.0)
**Pre- and Post-Treatment Tumor Dimensions**	
Preoperative Max Diameter (mm)	26.0 (22.5–28.0)
Preoperative Tumor Volume (mm^3^)	5737.2 (2374.3–7961.2)
Pre-Medical Therapy Max Diameter (mm)	16.0 (3.0–24.7)
Pre-Medical Therapy Tumor Volume (mm^3^)	1514.2 (14.0–3705.0)
Post-Medical Therapy Max Diameter (mm)	7.8 (2.6–13.4)
Post-Medical Therapy Tumor Volume (mm^3^)	182.5 (9.5–645.6)
Delta Max Diameter Pre-to-Post Medical Therapy (mm)	-10.1 (-15.0 - -2.6)
Delta Tumor Volume Pre-to-Post Medical Therapy (mm^3^)	-1450.1 (-3177.2 - -575.9)
Percent of Tumor Volume Reduction from Pre-to-Post Medical Therapy	87.9%

### Targeted therapy type and dosing regimen

Most patients (14/19, 73.7%) received dual BRAF and MEK inhibitor therapy, while four (21.1%) received BRAF inhibitor monotherapy, and one (5.3%) MEK inhibitor monotherapy (Table 1). BRAF inhibitor agents included Vemurafenib (7 patients), Dabrafenib (5 patients), and Encorafenib (6 patients). MEK inhibitors were Trametinib (5 patients), Cobimetinib (5 patients), and Binimetinib (5 patients). Dosing regimens for each agent are individually detailed in Table 1. Targeted therapy was initiated a median of 3.0 months (2.0–5.5) after surgical resection, and patients completed a median of 4.0 (range: 1–26) cycles of therapy (median cycle length = 28 days).

### Targeted therapy-related outcomes

**Fig. 2:**
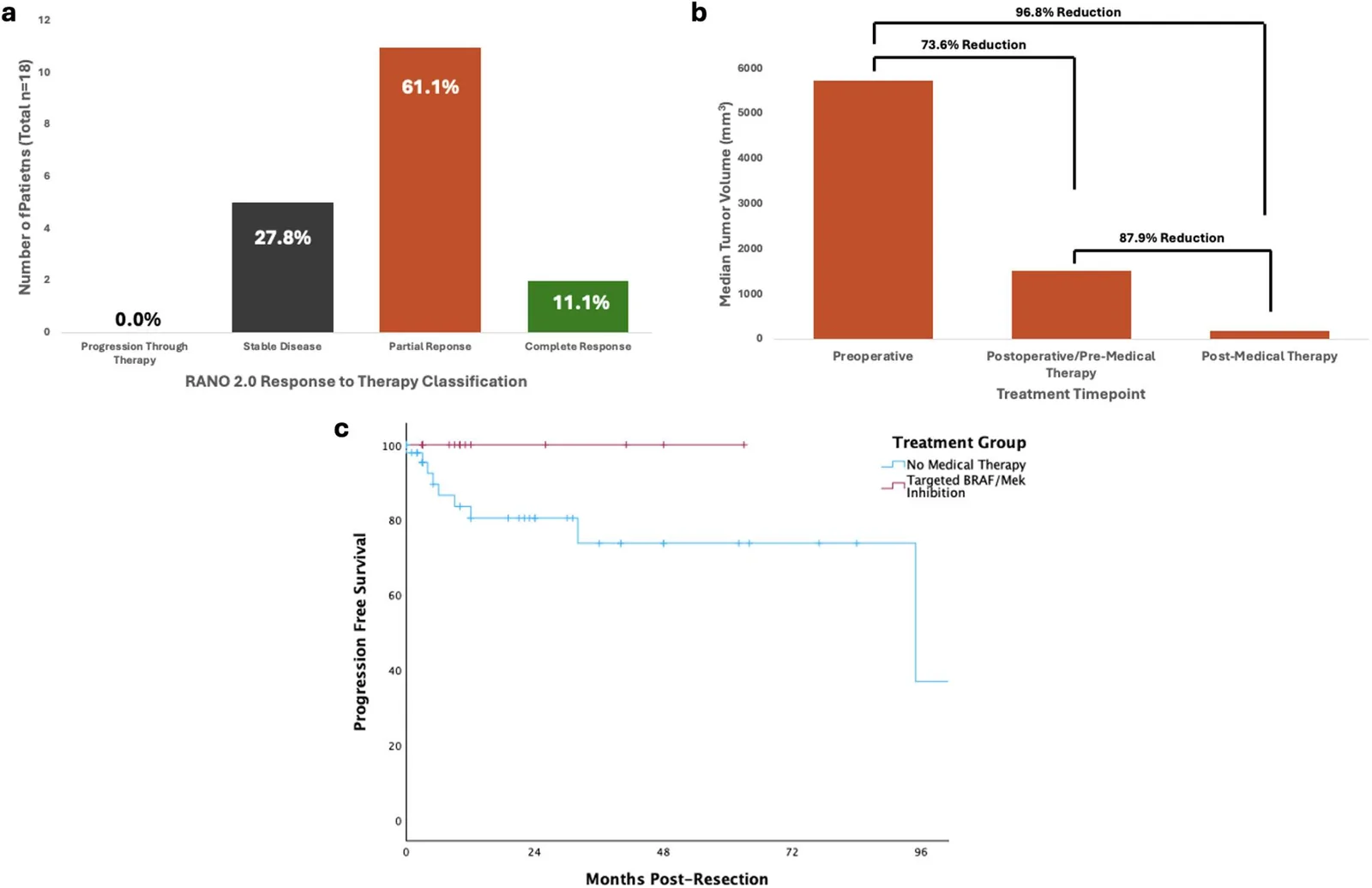
Panel **A** demonstrates the rates of each RANO 2.0 response class among 18 included patients with sufficient follow-up. Panel **B** shows the progression of median tumor volume of the cohort preoperatively, postoperatively before the initiation of medical therapy, and after the completion of medical therapy. Panel **C** presents a Kaplan-Meier curve comparing the median progression free survival of the included cohort of PCP patients treated with BRAF and MEK inhibition (*n* = 18) to a control cohort of 57 PCP patients who did not receive medical therapy

Eighteen patients had sufficient follow-up, defined as a minimum of 1 month follow-up after the completion of targeted therapy with available post-therapy MRI data, to assess their response to targeted therapy (Table 1**)**. Two patients (11.1%) demonstrated a complete response, 11 (61.1%) a partial response, five (26.3%) had stable disease, and none (0.0%) showed progressive disease through therapy (Figs. 1B and 2A). Nine patients (47.4%) stopped medical therapy due to AEs. Grade 1 and 2 AEs were most common (11, 57.9%), followed by none (3, 15.8%), Grade 3 (3, 15.8%), and Grade 4 (1, 5.3%). No Grade 5 AEs were noted. Specific AEs observed are detailed in **Supplemental Table 1**. Three additional patients eventually stopped therapy due to patient or provider preference unrelated to specific AEs. Tumor volume decreased by a median of 1450.1 mm^3^ (575.9–3177.2 mm^3^), from pre- to post-medical therapy (Fig. 2B).

### Radiation-sparing strategy

Fourteen patients did not receive any radiation during their medical therapy treatment course and did not demonstrate progressive disease. Four patients in the cohort received adjuvant radiation, two before medical therapy, and two after therapy cessation, despite no progression. Medical therapy treatment regimens were similar, except for one patient receiving adjuvant radiation and MEK inhibitor monotherapy (Table 2). Overall responses to therapy (*p* > 0.07) (rate of partial response = 7/14 in radiation-naïve group versus 4/4 in radiation group; complete response = 2/14 in radiation-naïve group, 0/4 in radiation group), medical therapy discontinuation due to AEs (radiation-naïve: 7/13, radiation: 2/4; *p* > 0.9), and AE grades were similar (*p* > 0.4). Radiation naive patients demonstrated lower median residual tumor volume after completion of medical therapy (23.7 vs. 612.4 mm^3^, *p* = 0.040), though while statistically insignificant, baseline tumor size trended larger in the radiation group. No other radiographic variables differed between the groups.

**Table 2:** Comparison of medical therapy regimen and outcomes between those who did versus did not undergo adjuvant radiation

Absolute # (%) or Median (IQR)	No Radiation (*n* = 15)	Adjuvant Radiation (*n* = 4)	*p*-Value
Medical Therapy Regimen			
BRAF Inhibitor Monotherapy	4 (26.7)	0 (0.0)	0.245
BRAF/MEK Inhibitor Dual Therapy	11 (73.3)	3 (75.0)	0.946
Time From Surg to Med Tx (mos)	3.00 (1.8–4.0)	7.00 (2.0–12.0)	0.425
Number of Cycles of Med Tx	4.00 (3.00–15.5)	7.50 (3.5–11.5)	0.920
**RANO Response to Therapy**	*N* = 14	*N* = 4	
Complete Response to Med Tx	2 (14.3)	0 (0.0)	0.423
Partial Response to Med Tx	7 (50.0)	4 (100.0)	0.070
Stable Disease Through Med Tx	5 (35.7)	0 (0.0)	0.160
**Cessation of Therapy and Adverse Events**			
Stop Medical Tx due to Adverse Event	7 (46.7)	2 (50.0)	0.906
Stop Medical Tx due to Progression	0 (0.0)	0 (0.0)	1.000
Stop Medical Tx for Other Reasons	1 (6.7)	2 (50.0)	0.035
**Grade of Adverse Events**			
Grade 1 Adverse Event	3 (20.0)	2 (50.0)	0.409
Grade 2 Adverse Event	5 (33.3)	1 (25.0)	0.475
Grade 3 Adverse Event	2 (13.3)	1 (25.0)	0.770
Grade 4 Adverse Event	1 (6.7)	0 (0.0)	0.533
Grade 5 Adverse Event	0 (0.0)	0 (0.0)	1.000
**Pre- and Post-Treatment Tumor Dimensions**			
Pre-Medical Therapy Max Diameter (mm)	16.0 (2.3–20.0)	22.3 (18.9–25.0)	0.169
Pre-Medical Therapy Tumor Volume (mm^3^)	1086.8 (7.8–2609.6)	3429.2 (2992.7–4013.7)	0.104
Post-Medical Therapy Max Diameter (mm)	4.00 (1.8–9.3)	13.2 (11.0–14.9)	0.056
Post-Medical Therapy Tumor Volume (mm^3^)	23.7 (3.5–208.3)	612.4 (421.45–821.2)	**0.040**
Delta Max Diameter Pre-to-Post Medical Therapy (mm)	-8.50 (-15.0 - -1.7)	-10.1 (10.5 - -8.6)	0.944
Delta Tumor Volume Pre-to-Post Medical Therapy (mm^3^)	-990.1 (-2625.6 - -192.7)	-2909.4 (-3338.3–2518.0)	0.142

### Monotherapy versus dual therapy

Four patients received BRAF inhibitor monotherapy, while 14 received BRAF and MEK inhibitor dual therapy **(**Table 3**).** One patient received MEK inhibitor monotherapy and was excluded from this analysis. BRAF inhibitor agents used and the time to initiation of medical therapy (*p* = 0.364) were similar between groups. BRAF inhibitor monotherapy patients received more therapeutic cycles (7.5 vs. 4.0, *p* = 0.521) than dual therapy patients, though not significant.

A higher rate of RANO 2.0 complete response was observed in the BRAF inhibitor monotherapy group (*n* = 2, 50.0% vs. *n* = 0, 0.0%, *p* = 0.007), though partial response (25.0% vs. 69.2%, *p* = 0.116) and stable disease (25.0% vs. 30.8%, *p* = 0.825) were similar. The rate of therapy discontinuation due to AEs was larger in the dual therapy group (25.0% vs. 57.1%, *p* = 0.257), but did not reach statistical significance. Individual AE grades did not differ between groups. However, no patients in the monotherapy group experienced Grade 3 or higher AEs, compared to four patients (28.5%) in the BRAF and MEK inhibitor dual therapy group. Median post-medical therapy volume (0.0 mm^3^ vs. 258.27 mm^3^, *p* = 0.011) was significantly less in the monotherapy cohort, baseline tumor size trended larger in the dual therapy group.

**Table 3:** Comparison of medical therapy regimen and outcomes between those treated with BRAF inhibitor monotherapy vs. dual BRAF/MEK inhibition

Absolute # (%) or Median (IQR)	BRAF Monotherapy (*n* = 4)	BRAF/MEK Dual Therapy (*n* = 14)	*p*-Value
Treatment Details			
Adjuvant Radiation	0 (0.0)	3 (21.4)	0.310
**Medical Therapy Regimen**			
Time From Surg to Med Tx (mos)	4.0 (3.5-4.0)	2.5 (1.8–3.8)	0.364
Number of Cycles of Med Tx	7.50 (2.5–15.5)	4.0 (3.0–13.3)	0.521
**RANO Response to Therapy**	*N* = 4	*N* = 13	
Complete Response to Med Tx	2 (50.0)	0 (0.0)	**0.007**
Partial Response to Med Tx	1 (25.0)	9 (69.2)	0.116
Stable Disease Through Med Tx	1 (25.0)	4 (30.8)	0.825
**Cessation of Therapy and Adverse Events**			
Stop Medical Tx due to Adverse Event	1 (25.0)	8 (57.1)	0.257
Stop Medical Tx due to Progression	0 (0.0)	0 (0.0)	1.000
Stop Medical Tx for Other Reasons	0 (0.0)	3 (21.4)	0.310
**Grade of Adverse Events**			
Grade 1 Adverse Event	1 (25.0)	3 (21.4)	0.469
Grade 2 Adverse Event	1 (25.0)	5 (35.7)	0.825
Grade 3 Adverse Event	0 (0.0)	3 (21.4)	0.425
Grade 4 Adverse Event	0 (0.0)	1 (7.1)	0.672
Grade 5 Adverse Event	0 (0.0)	0 (0.0)	1.000
**Pre- and Post-Treatment Tumor Dimensions**			
Pre-Medical Therapy Max Diameter (mm)	1.50 (1.4–11.3)	16.0 (14.0–22.3)	0.309
Pre-Medical Therapy Tumor Volume (mm^3^)	1.53 (1.26–1950.26)	1514.24 (992.68–3497.06)	0.484
Post-Medical Therapy Max Diameter (mm)	0.00 (0.0–0.7)	9.3 (4.75–12.7)	**0.011**
Post-Medical Therapy Tumor Volume (mm^3^)	0.00 (0.0–0.2)	258.27 (57.7–573.2)	**0.011**
Delta Max Diameter Pre-to-Post Medical Therapy (mm)	-1.31 (-11.6 - -0.71)	-10.8 (14.5 - -4.4)	0.612
Delta Tumor Volume Pre-to-Post Medical Therapy (mm^3^)	-1.09 (-1950.0 - -1.04)	-1450.1 (-3061.1–948.0)	0.499

### Short versus long course therapy

No significant differences in BRAF/MEK inhibitor agents or time to initiation of therapy (*p* = 0.687) were observed between those who completed four or fewer cycles (short course, *n* = 7) versus those who completed more than four cycles (long course, *n* = 12) (**Supplemental Table 2**). The long course group achieved higher rates of RANO 2.0 partial response to therapy (81.8% vs. 28.6%, *p* = 0.024). Rates of complete response were similar (9.1% vs. 14.3%, *p* = 0.732). No differences were noted in rates of therapy discontinuation due to AEs (*p* = 0.515), grades of AE, or radiographic outcomes.

### Progression free survival

Median PFS in the targeted therapy group (*n* = 16) was not reached, since no patients progressed during the study. In comparison, PFS in the control group (*n* = 57) was 5.0 months (range, 1.0–95.0 months), though this difference did not reach statistical significance (*p* = 0.108). A Kaplan-Meier curve illustrating the comparison is presented in Fig. 2C. No patients were deceased during the study period.

## Discussion

### Previous study

While the strong correlation of the BRAF V600E mutation with PCP was described a decade ago, the initial implementation of mutation specific targeted therapies was limited to small case series [3, 9]. Finally, the first phase I/II clinical trial provided early evidence of safety and efficacy for the use of vemurafenib and cobimetinib in the treatment of PCP [8]. The early trial data supports a major paradigm shift in treatment of PCP; however, the real-world implementation of targeted inhibition remains unclear and consensus standard of care protocols or optimal treatment regimens are lacking. Here, leveraging the RAPID Consortium, we describe differing practice patterns across the 8 participating skull base programs and how they may impact outcomes, potentially informing the design of future studies.

### Current study

Consistent with previous studies, our cohort demonstrates excellent rates of complete or partial response with no cases of progression during the 12-month median follow-up period. Specifically, we noted 11 partial responses, 2 complete responses and the remainder showed stable disease. Like prior clinical trials, adjuvant therapy was well tolerated, with patients completing a median of 4.0 cycles and experiencing primarily no or Grade 1/2 CTCAE (79%). Rate of therapy discontinuation due to AEs was higher in the presented cohort compared to the phase 2 Alliance A071601 trial for BRAF and MEK inhibition in PCP. Despite higher rates of grade 3 and 4 AEs in the trial, only 3 patients discontinued therapy early, which likely reflects more stringent criteria in the trial guidelines compared to the RAPID cohort for interruption of protocol therapy. Following BRAF and MEK inhibitor initiation, additional volumetric tumor reduction was evident in most cases on MR imaging. In this multi-institutional cohort, we noted three main practice patterns: (1) radiation-sparing strategy, (2) monotherapy versus dual therapy, and (3) short versus long treatment course.

### Radiation-sparing treatment

The modern treatment paradigm for craniopharyngioma in adults, irrespective of type, is pursuit of maximal safe resection with adjuvant radiation in the setting of a subtotal resection. This achieves similar local control rates, and decreased adverse effects compared to upfront gross total resection [1, 19–22]. Conversely, Damante and colleagues previously postulated the potential for a radiation-sparing strategy following subtotal resection of PCPs, given the expected response to targeted therapies [4]. While radiation may assist in disease control, damage to neuro-endocrine structures results in new or progressive endocrinopathy in up to 94% of patients over 5 years [20, 21]. Furthermore, 11% of adults with craniopharyngioma have been shown to develop grade two vision deterioration after radiotherapy, with a similar proportion developing symptomatic memory impairment [23, 24]. 

In the present cohort, only four patients were treated with radiation after the index surgery. Two of these patients underwent radiation in an adjuvant manner after the index resection and later received targeted therapy due to an incomplete response to radiation. The remaining two cases underwent radiation after targeted therapy cessation, one after therapy was stopped due to an AE, and the other intentionally trialed a short course of medical therapy before planned radiation 1 month later. It is unclear if adjuvant radiation provided additional local control benefit. Patients that did not receive radiation had a smaller residual tumor volume after medical therapy completion, though the cohort receiving radiation had slightly, but not statistically significant, larger pre-treatment tumor size. The patients who did not receive radiation demonstrated similar rates of disease control on medical therapy alone, though these comparisons should be interpreted cautiously and considered as hypothesis generating due to the particularly small size of the radiation group in this cohort. Recent studies have observed similar outcomes with radiation-sparing protocols [4, 10, 11]. Though, the possibility of selection bias must also be noted here as radiation therapy was used at the discretion of each participating RAPID site and eventual outcomes may have been influenced by clinicians favoring radiation-sparing approaches for cases with smaller lesions, lower neuro-endocrinologic symptom burden, or other patient-specific factors that could not be accounted for in this retrospective study.

### Monotherapy treatment

The combination of BRAF and MEK inhibitor therapy in malignancy is employed to mitigate the potential risk of therapeutic resistance, as described in melanoma [5, 6]. BRAF monotherapy results in reactivation of the ERK pathway due to reduced negative feedback regulation from up or downstream of BRAF [11, 25–28]. This risk seems plausible in a rapidly dividing, malignant cell state, like melanoma [6, 26]. Rather, PCP is a benign lesion with much more genetic stability [3, 8, 13, 29]. Therefore, the risk of therapeutic resistance seems less pressing, though has not been studied.

In the present cohort, monotherapy appeared at least non-inferior to dual therapy, though dual therapy was most often employed. There were four instances in which BRAF monotherapy was initiated, thus, all statistical comparisons should be interpreted with caution. Overall, RANO response rates and volumetric tumor reduction with monotherapy versus dual therapy were similar between groups. There are individual case reports depicting similar radiographic control with BRAF monotherapy on extended treatment courses of 1- to 2-years and no reported AEs [27, 28]. Alternatively, in pediatric low-grade glioma, a BRAF altered tumor more similar to PCP than melanoma due to its indolent biology, dual therapy has often been shown to be more efficacious than monotherapy. With the small sample size and lack of pre- or post-treatment molecular/genetic data for the included cohort, it is difficult to make mechanistic assumptions as to why BRAF monotherapy showed greater success in this PCP cohort than prior glioma studies and should be a focus of future basic science investigations on the topic. We suspect the changes are due to fewer mechanistic drivers in PCP making the disease more treatable with targeted therapy.

Comparing the two groups, there were fewer observed overall CTCAE, with no cases of grade 3 or higher AEs in the monotherapy group, despite slightly longer treatment courses. Similarly, only one patient had to stop BRAF monotherapy due to an AE compared to eight in the dual therapy group. In melanoma, BRAF and MEK inhibitor therapy is associated with fewer complications, however, the most frequently reported AEs from BRAF monotherapy are skin-related changes (CTCAE Grade 1–2) [6, 30]. The addition of MEK inhibitors subjected patients to higher rates of specific MEK-class toxicities, including cardiotoxicity (which may be reversible upon discontinuation), but overall similar rates of grade 3 and 4 CTCAEs to BRAF monotherapy [30, 31]. Other PCP case reports utilizing BRAF monotherapy have not observed any AEs at more than 1-year of treatment [4, 27, 28, 32]. One patient in the presented cohort alternatively received MEK inhibitor monotherapy, resulting in a partial response to therapy with only a Grade 1 AE that did not require therapy cessation. However, with only one such patient it is difficult to draw any conclusions regarding the effectiveness of MEK inhibitor monotherapy relative to BRAF monotherapy or BRAF/MEK dual therapy.

### Treatment duration

BRAF and MEK inhibitor therapy is used in the treatment of multiple malignancies. The most common therapeutic combination is dabrafenib 150 milligrams twice daily and trametinib 2 milligrams daily, though there are others, such as vemurafenib 960 milligrams twice daily with cobimetinib 60 milligrams daily in the PCP trial. This variability in dosing regimens between approved therapies accounts for the variability in dose of BRAF and/or MEK inhibitors received by each patient [8, 30, 33–36]. Across these clinical trials, the number of 3-to-4-week treatment cycles for BRAF and MEK inhibitor therapy was approximately 8 or more (the single PCP-specific trial evaluated 4 cycles) [8, 30, 33–36]. The dosing and duration of treatment employed in these studies originate largely from the early melanoma dose escalation studies, though titration was not targeted for CNS penetration [37–39]. 

In the presented cohort, a median of four therapeutic cycles were employed, therefore we stratified our analysis by patients receiving four or less cycles compared to more than four cycles of therapy. A higher rate of partial radiographic response was observed in patients treated with the longer course. There were similar rates of CTCAE observed regardless of a short or long course of therapy. Conversely, a recent large retrospective cohort suggested the greatest response to therapy was observed within one month, with additional, albeit less significant degree of response at three months [11]. 

### Limitations

This study represents a multi-institutional cohort of PCPs treated at US skull base centers and has several limitations. First, the retrospective design is subject to treatment selection bias that may drive decisions such as the use of radiation or monotherapy versus dual therapy and incomplete medical records. Moreover, the retrospective ascertainment of AEs is particularly subject to error. Second, there is unavoidable temporal bias given the rapid acceptance and use of targeted therapy for PCP that is evolving alongside advances in surgical technique and radiation therapies. Third, the relatively short median follow-up period of 12 months limits interpretations regarding the long-term durability of these results and true value of targeted therapy as a potential radiation-sparing strategy for these patients long-term. Finally, given the rarity of the diagnosis and lack of standard approach for BRAF and MEK inhibitors in PCP, the sub-analyses are underpowered. Each approach should be considered independently, as sub-analyses of combined algorithms was not possible. However, the aim of the study is not to conclude superiority of a certain approach – and statistically significant results should be interpreted cautiously and considered hypothesis-generating – but to explore the current practice patterns and suggest important considerations for future study. Detailed evaluation of neuro-endocrinologic outcomes after the implementation of medical therapy are also lacking from this study and should be a focus of future work.

Only 19 of 124 PCP patients in the RAPID database received targeted therapy at some point during their management. Given that these sites are all specialized centers with access to state-of-the art care, this highlights how rare targeted therapy use for PCP remains in current practice. Much of this low number can likely be attributed to this database spanning back through 2017, and the implementation of targeted therapy being relatively novel. However, while the reported cases were relatively evenly spread over the 8 participating centers, these centers represent less than half of active RAPID sites, with the remaining having no qualifying cases – demonstrating that this technique has still not been implemented on a broad scale.

#### Future directions

Important future directions for this work primarily will revolve around better characterizing the most optimal dosing regimen for each individual patient, centered around more detailed analysis of the main practice patterns identified in the presented work. Additional considerations should consist of the optimal treatment duration based on initial response. Each of these factors will likely be altered by tumor size, location, and other patient specific factors that will require significantly larger datasets to appropriately tease through. Finally, future studies with longer median follow up may be able to further elucidate the long-term efficacy of targeted therapy in PCP and rates of tumor recurrence or rebound effects after therapy cessation.

## Conclusion

In this multi-institutional cohort, BRAF and MEK-targeted therapy for papillary craniopharyngioma demonstrated high radiographic response rates and favorable short-term disease control. Variability in treatment strategy was observed, including radiation-sparing strategies, monotherapy use, and differences in treatment duration. These new therapeutic approaches appear safe and feasible, warranting further investigation. Given the rarity of the diagnosis, future prospective study leveraging multi-institutional consortia would allow for an expedited approach to understanding the optimal application of targeted therapies for craniopharyngioma.

## Supplementary Information

Below is the link to the electronic supplementary material.


Supplementary Material 1


## Data Availability

The datasets generated during and/or analyzed during the current study are available from the corresponding author on reasonable request.
