# Supplementary material for: Molecular therapy for papillary craniopharyngioma: a multi-institutional analysis of practice patterns across the RAPID Consortium
Source: J Neurooncol. 2026 Aug 14;179(1):40. doi: 10.1007/s11060-026-05739-5 (PMC13476336; doi:10.1007/s11060-026-05739-5)
Supplement: Supplementary file 1 [file 11060_2026_5739_MOESM1_ESM.pdf]

Supplemental Information

Supplemental Table 1. Specific Adverse Events Reported By Grade

AE Grade	Adverse Events
**Grade 1**	Rash/Skin-Changes (2); Fatigue/Weakness (2); Mildly Decreased EF (1); Vomiting/Diarrhea (1)
**Grade 2**	Rash/Skin-Changes (5); Vomitting/Diarrhea (1); Fever (1); Uveitis (1); Tongue Leukoplasia/Dysplasia (1)
**Grade 3**	Hyperglycemia (1); Elevated Liver Enzymes (1); Fevers/Chills/Myalgias Requiring Hospitalization (1); Vomitting/Diarrhea (1); Rash/Skin Changes (1)
**Grade 4**	Elevated Creatinine (1); Severe Edema (1)

**Supplemental Table 2. Comparison of Medical Therapy Regimen and Outcomes Between Those Treated with Under 4 Cycles versus 4+ Cycles of Targeted Therapy**			
Absolute # (%) or Median (IQR)	**Under 4 Cycles of Med Tx (n=7)**	**4+ Cycles of Med Tx (n=12)**	**p-Value**
**Treatment Details**			
Adjuvant Radiation	1 (14.3)	3 (25.0)	0.581
**Medical Therapy Regimen**			
BRAF/MEK Dual Therapy	5 (71.4)	9 (75.0)	0.865
Time from Surgery to Med Tx (mos)	3.50 (2.75 – 4.50)	3.00 (1.75 – 5.25)	0.667
**RANO Response to Therapy**	**N=7**	**N=11**	
Complete Response to Med Tx	1 (14.3)	1 (9.1)	0.732
Partial Response to Med Tx	2 (28.6)	9 (81.8)	**0.024**
Stable Disease Through Med Tx	4 (57.1)	1 (9.1)	**0.026**
**Cessation of Therapy and Adverse Events**			
Stop Medical Tx due to Adverse Event	4 (57.1)	5 (41.7)	0.515
Stop Medical Tx due to Progression	0 (0.0)	0 (0.0)	1.000
Stop Medical Tx for Other Reasons	2 (28.6)	1 (8.3)	0.243
**Grade of Adverse Event**			
Grade 1 Adverse Event	2 (28.6)	3 (25.0)	0.699
Grade 2 Adverse Event	3 (42.9)	3 (25.0)	0.264
Grade 3 Adverse Event	1 (14.3)	2 (16.7)	0.923
Grade 4 Adverse Event	0 (0.0)	1 (8.3)	0.464
Grade 5 Adverse Event	0 (0.0)	0 (0.0)	1.000
**Pre- and Post-Treatment Tumor Dimensions**			
Pre-Medical Therapy Max Diameter (mm)	3.00 (2.16 – 11.45)	17.5 (15.0 – 24.8)	0.277
Pre-Medical Therapy Tumor Volume (mm^3)	14.0 (7.51 – 1651.6)	1808.8 (1039.7 – 3746.0)	0.295

Post-Medical Therapy Max Diameter (mm)	3.00 (1.5 – 5.8)	10.0 (4.0 – 13.8)	0.185
Post-Medical Therapy Tumor Volume (mm^3)	14.0 (7.0 – 148.3)	234.0 (22.9 – 682.8)	0.311
Delta Max Diameter Pre-to-Post Medical Therapy (mm)	-1.31 (-6.3 - -0.66)	-10.2 (-15.0 - -4.60)	0.161
Delta Tumor Volume Pre-to-Post Medical Therapy (mm^3)	-0.98 (-1503.8 - -0.49)	-1635.6 (-3275.1 - -990.1)	0.170
